# Heat stress decreased transpiration but increased evapotranspiration in gerbera

**DOI:** 10.3389/fpls.2023.1119076

**Published:** 2023-01-19

**Authors:** Zaiqiang Yang, Yuhan Jiang, Rangjian Qiu, Xuewen Gong, Evgenios Agathokleous, Wei Hu, Brent Clothier

**Affiliations:** ^1^ Jiangsu Key Laboratory of Agricultural Meteorology, School of Applied Meteorology, Nanjing University of Information Science and Technology, Nanjing, China; ^2^ State Key Laboratory of Water Resources and Hydropower Engineering Science, School of Water Resources and Hyperpower Engineering, Wuhan University, Wuhan, China; ^3^ School of Water Conservancy, North China University of Water Resources and Electric Power, Henan Key Laboratory of Crop Water Use, Zhengzhou, China; ^4^ The New Zealand Institute for Plant and Food Research Ltd, Christchurch, New Zealand; ^5^ The New Zealand Institute for Plant and Food Research Ltd, Auckland, New Zealand

**Keywords:** high temperature, leaf stomata, root activity, stomatal conductance, transpiration, evaporation

## Abstract

Heat stress is a major constraint for plant production, and evapotranspiration is highly linked to plant production. However, the response mechanism of evapotranspiration to heat stress remains unclear. Here, we investigated the effects of heat stress during two main growth stages on transpiration and evapotranspiration of gerbera. Two levels of day/night temperature were adopted during the vegetative growth stage (VG) and the flowering bud differentiation stage (FBD), namely control (CK; 28/18 °C) and heat stress (HS; 38/28°C) levels. The duration of HS was set as 5, 10, 15, and 20 days, respectively. At the beginning of HS, hourly transpiration was mainly inhibited near noon. With continuation of HS, the duration and extent of inhibition of hourly transpiration increased. Daily transpiration rate was also markedly reduced by HS during the VG (18.9%-31.8%) and FBD (12.1%-20.3%) stages compared to CK. The decrease in the daily transpiration rate was greater for longer duration of heat stress. This reduction of transpiration was the main contributor to stomatal limitation at the beginning of HS, while additional inhibition of root activity, leaf area, and root biomass occurred under long-term HS. The daily transpiration rate could not recover after the end of HS (so-called recovery phase), except when HS lasted 5 days during the VG stage. Interestingly, daily evapotranspiration during HS was substantially increased during the VG (12.6%-24.5%) and FBD (8.4%-17.6%) stages as a result of more increased evaporation (100%-115%) than reduced transpiration. However, during the recovery phase, the daily evapotranspiration was markedly decreased at the VG (11.2%-22.7%) and FBD (11.1%-19.2%) stages. Hence, we suggest that disproportionate variation of transpiration and evaporation during HS, especially at the recovery phase, should be considered in various evapotranspiration models and climate scenarios projections.

## 1 Introduction

Evapotranspiration is the water transferred from the land surface to the atmosphere, which involves a phase change of water converted from liquid to water vapor (Wang et al., 2012). The evapotranspiration is not only an important component in the water and energy balance sectors, but is also highly linked to plant growth, production of biomass and yield, and quality (Bastug et al., 2006; Katsoulas et al., 2006; Qiu et al., 2021; Qiu et al., 2022; Qin et al., 2023; Qiu et al., 2023). It also plays a critical role in land–atmosphere interactions in the earth system (Wang and Dickinson, 2012). The magnitude of evapotranspiration is affected by many factors, including meteorological conditions, soil and crop factors, and management and environmental conditions, such as water, salinity, and heat stresses (Allen et al., 1998). Many factors including water and salinity stresses affecting evapotranspiration have been well described in numerous studies (Allen et al., 1998; Qiu et al., 2017; Minhas et al., 2020; Yang et al., 2020). However, effect of heat stress on evapotranspiration deserves more attention as a result of global warming. More frequent and intense heat stress caused by global warming continues to draw research attention due to the great impacts on plant production. For a future 2.0°C warming level in a climate without human effect, the intensity of heat stress will increase 2.7°C and frequency will occur 13.9 times higher than 1850-1900 (IPCC, 2021). Heat stress is defined as a temperature rise above the threshold level, usually 10-15°C above the typical ambient temperature, which occurs on different time scales with different intensities and duration levels (Wollenweber et al., 2003; Teskey et al., 2015; Ishimaru et al., 2016). Heat stress can affect organisms directly or indirectly by changing the surrounding environmental components. Since plants cannot move to a more favorable environment, heat stress may severely affect plant growth and development, as well as evapotranspiration (Lobell and Asner, 2003; Lobell and Field, 2007).

The negative effects of heat stress on plant growth and production have been well described. Transient or persistent heat stress can induce morphological, physiological, and phenological responses in plants (Tubiello et al., 2007; Ahuja et al., 2010; Mittler and Blumwald, 2010; Hasanuzzaman et al., 2013). For instance, the effect of heat stress on plant morphology generally includes sunburn of stems, leaves and branches, premature senescence and abscission of leaves, inhibition of shoot and root growth, and discoloration and damage of fruits (Wahid et al., 2007; Lipiec et al., 2013; Hatfield and Prueger, 2015). Heat stress also reduces cell division and restricts cell elongation, resulting in retardation of plant growth (Ashraf and Harris, 2004; Camejo et al., 2005; Daly et al., 2004). In addition, heat stress can damage chloroplast and decrease photosystem II (PSII) activity and the number of photosynthetic pigments, and thus also negatively affect gas exchange and photosynthesis (Salvucci and Crafts-Brandner, 2004; Allakhverdiev et al., 2008). Moreover, heat stress can alter leaf water status and stomatal conductance, promote water transport, and reduce transpiration by reducing cell size, increasing xylem vessel diameter, and stomatal density (Guiguitant et al., 2017; Devi and Reddy, 2018).

However, the response mechanism of evapotranspiration to heat stress remains unclear. We hypothesize that heat stress has negative effects on transpiration because of adverse effects of heat stress on plant growth and production, while it has positive effects on evaporation as a result of increased vapor pressure deficit. This opposite influence of heat stress on transpiration and evaporation leads to an uncertain effect of it on evapotranspiration. In addition, the duration and the occurrence stage of heat stress may have different effects on transpiration and thereby evapotranspiration. Moreover, it is also unclear whether the transpiration and evapotranspiration could be recovered after the end of varying duration of heat stress (recovery phase). Hence, gerbera, an important commercial flower worldwide mainly cultivating in the protected agriculture (Wani et al., 2018; Darras, 2021), was used as an example crop in this study, which frequently suffer from heat stress. Our objectives were (1) to investigate the response of transpiration and evapotranspiration to varied duration of heat stress under the two main growth stages, (2) to explore the mechanism for heat stress induced variation of transpiration and evapotranspiration, and (3) to reveal the regulation of transpiration and evapotranspiration during recovery phase.

## 2 Materials and methods

### 2.1 Experimental details and plant materials

The experiment was conducted at the Agro-Meteorology Research Station of Nanjing University of Information Science and Technology, located at Nanjing, Jiangsu Province, China (32°13′ N, 118°41′ E, altitude 14.4 m) during December 2021 to March 2022. The top and bottom diameters of each pot were 21.0 and 16.5 cm, respectively, and the height was 21.0 cm. The soil substrate was a mixture (2:1) of peat and perlite, which is commonly used to cultivate gerbera in protected agriculture. The soil substrate was a mixture (2:1) of peat and perlite, which is commonly used to cultivate gerbera in protected agriculture. The diameter of fiber granules in the soil substrate ranges from 0 to 25 mm. The PH of this soil is 6.0. The soils contain fertilizers, including nitrogen (140 mg N L^-1^), phosphorus (100 mg P_2_O_5_ L^-1^), potassium (180 mg K_2_O L^-1^), magnesium (100 mg Mg L^-1^), and micronutrient (480 mg L^-1^). The water-retaining property of this soil is 75-80%. Before transplanting, the soil was saturated with fresh water and freely drain for 12h (covering with plastic mulch) to determine the water holding capacity of the pot (W_max_ = 0.35 kg). Gerbera plants (Chrysanthemum Morifolium, cultivar Rionegro) were transplanted to the pots when they formed five to six functional leaves. The transplanted plants were initially grown in a greenhouse to keep suitable growth condition, and then moved to artificial climate chambers (Convion BDW40, Canada). The artificial climate chamber had 3.05 m length, 1.78 m width, and 2.90 m height. The heat stress manipulation was imposed after 7 days of acclimation for the gerbera plants to the environment of the artificial climate chamber for both vegetative growth stage and flowering bud differentiation stage.

### 2.2 Experimental design

**Figure 1:**
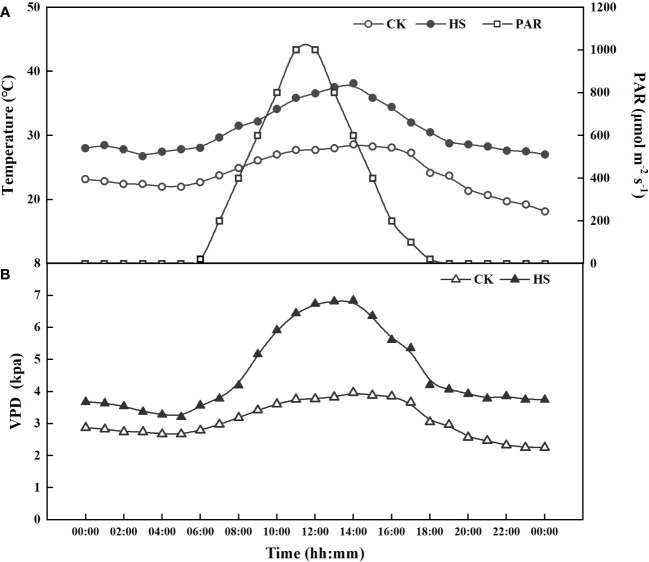
The actual photosynthetic active radiation (PAR), air temperature **(A)**, and vapor pressure deficit (VPD) **(B)** in CK and heat stress (HS) treatments in the artificial climate chambers.

In this study, heat stress experiments were carried out for two growth stages, i.e. the vegetative growth stage and the flowering bud differentiation stage. Two levels of day/night temperature were adopted: control level (CK; 28/18 °C) and heat stress level (HS; 38°C/28°C) (Kempkes and Van de Braak, 2000; Tsirogiannis et al., 2010; Janka et al., 2013). The duration of heat stress was set as 5, 10, 15, and 20 days, respectively, resulting in 8 conditions for each growth stage (**Table 1**). For each condition, there were 9 pots. After the end of the heat stress period, the pots were moved to the artificial climate chambers of CK for recovery (recovery phase). All the plants were irrigated daily at 18:00 to reach 95% of Wmax. The day/night temperature represents the highest and lowest temperature during the day. The diurnal variation of air temperature in the artificial climate chamber was set to be similar to that observed in Nanjing city, with the minimum and maximum temperatures appearing at 5:00 and 14:00, as shown in **Figure 1A**. Overall, the actual values of air temperature in the artificial climate chamber were slightly higher than the setting values, especially near noon. The actual maximum and minimum temperatures were 38.13 ± 0.55 and 28.30 ± 0.48°C for HS treatment, and 28.56 ± 0.23 and 19.25 ± 0.07°C for CK treatment.

**Table 1:** Experimental design of heat stress under two growth stages. V and F indicate the vegetative growth stage and flowering bud differentiation stage.

Experiment 1: Vegetative growth stage		Experiment 2: Flowering bud differentiation stage	
Treatment	Duration	Treatment	Duration
CK		CK	
HS-V-5	5	HS -F-5	5
HS-V-10	10	HS -F-10	10
HS-V-15	15	HS -F-15	15
HS-V-20	20	HS -F-20	20

The light and relative humidity were the same for all the treatments. The light was supplemented from 6:00 to 19:00 (day time), with a maximum photosynthetic active radiation of 1000 μmol m^-2^ s^-1^ (**Figure 1A**). The relative humidity (RH) was set at 40 ± 5% and 60 ± 5%, respectively, for day and night. The hourly vapor pressure deficit (VPD=*e_s_*-*e_a_*) can be calculated based on measured air temperature and RH, as (Allen et al., 1998):


(1) $$e_{s}=0.6108exp[\frac{17.27T}{T+237.3}]$$

(2) $$e_{a}=e_{s}\frac{RH}{100}$$

where *e_s_* and *e_a_* are the saturation and actual vapor pressure (kPa), respectively. There were great differences in the daytime VPD between the heat stress and CK treatments, with a maximum value of 2.31 kPa near noon (**Figure 1B**).

### 2.3 Measurements

#### 2.3.1 Root activity

Root activities of three plants for each treatment were determined by the triphenyl tetrazolium chloride (TTC) reduction method (Onanuga and Adl, 2012). A spectrophotometer was used to determine the absorbance values of different concentrations of TTC solutions at λ = 485 nm to establish a standard curve, which was then used to calculate the reduction intensity of TTC as (Onanuga and Adl, 2012):


(3) $$TTC\;reduction\;intensity=\frac{TTC\;reduction\;mass}{fresh\;mass\;of\;root\times time}$$

#### 2.3.2 Root dry mass and leaf area

Three randomly selected plants for each treatment were used for measuring root dry mass. The roots were washed with fresh water, and excess water in the roots was absorbed by filter paper. Then the fresh masses of roots were measured with a precision electronic scale with an accuracy of 0.001g. After that, the dry masses of roots were determined after oven-drying at 80°C.

The leaf area of three plants for each treatment was measured with a LAI 3000 leaf area areometer (Li-Cor Inc., USA) every 5 days after plants were transferred into the artificial climate chamber.

#### 2.3.3 Leaf stomatal length, width, and density

Stomatal properties of leaves were determined by using the imprinting method (Radoglou and Jarvis, 1990). Five functional leaves of the plants were randomly sampled during 10:00-11:00. After wiping off the dust from the leaf surface with a skimmed cotton pad, nail polish was applied to the middle of the leaf between the center of the veins and the leaf edge. The film with imprint was then extracted after nail polish dried and used as samples.

The stomata of leaf epidermal cells for each leaf were then observed by using a light microscope (Olympus CX-31, China) at 40x. With the use of a combined digital imaging system (Olympus dp-20, China), the stomatal density could be determined after measuring the average number of stomata in 15 visual fields. The stomatal length and width of 10 visual fields were then calibrated by using a digital ranging software (Motic Images Advanced 3.0, China), and then mean values were used.

#### 2.3.4 Leaf stomatal conductance and leaf transpiration

Gas exchange, including leaf stomatal conductance and leaf transpiration, were measured in three randomly selected plants by a LI-6400XT photosynthesis system (LI-COR Inc., Lincoln, NE, USA) during 9:00-11:00. The photosynthetic active radiation was fixed at 1000 μmol m^-2^ s^-1^, CO_2_ was fixed to 400 μmol mol^-1^, and flow rate was 500 μmol s^-1^.

#### 2.3.5 Transpiration and evapotranspiration

The transpiration and evapotranspiration were determined based on water balance method after the plants were moved into the artificial climate chambers. To measure the transpiration rate, the soil surface of three pots for each treatment was covered with black plastic film to avoid evaporation. The evaporation rate was determined as the difference between evapotranspiration and transpiration. In addition, the hourly transpiration and evapotranspiration were determined every two hours by using the water balance method (Qiu et al., 2015; Li et al., 2018).

### 2.4 Data analysis

A general linear model-univariate procedure was used to carried out two-way analysis of variance (ANOVA) using SPSS software (Version 23, IBM Corp., USA). ANOVA was used to determine the effects of heat stress, growth stages, and their interactions on root actively, root dry mass, leaf area, leaf stomatal characteristics, leaf stomatal conductance, transpiration, and evapotranspiration. All the treatment means were compared at a significance level of *P* ≤ 0.05.

## 3 Results

### 3.1 Effect of heat stress on root activity and root dry mass

**Figure 2:**
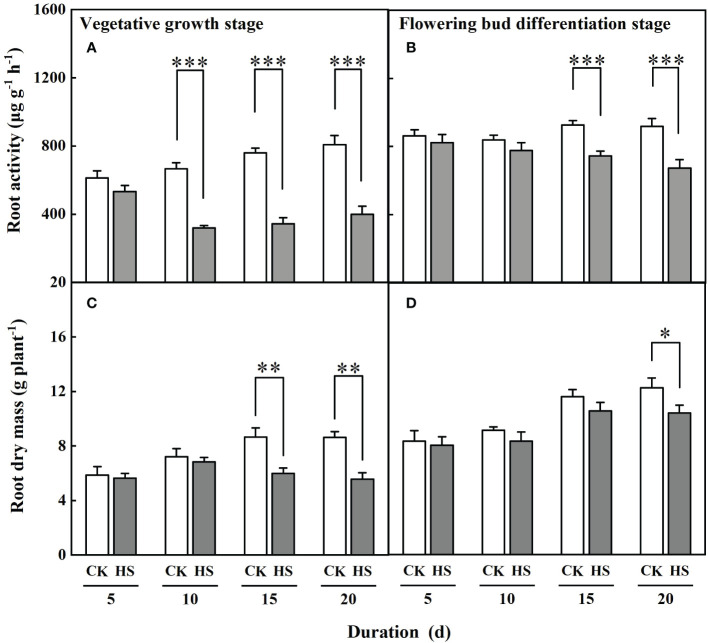
The root activity **(A, B)** and dry mass **(C, D)** of gerbera under different temperature treatments during the vegetative growth **(A, C)** and flowering bud differentiation stages **(B, D)**. CK is the control treatment, HS is the heat stress treatment. Values are the mean ± SD (n=3). *, **, and *** represent the significant level at *P*<0.05, *P*<0.01, and *P*<0.001, respectively.

**Figure 2** shows that the values of root activity of gerbera were significantly decreased after a 10-day-long heat stress during the vegetative growth stage and a 15-day-long heat stress during the flowering bud differentiation stage. Compared to CK, the root activity of gerbera was reduced by 51.9%, 54.8%, and 50.6%, respectively, for the HS-V-10, HS-V-15, and HS-V-20 conditions, and by 19.3% and 26.8% for HS-F-15 and HS-F-20 conditions. ANONA analysis also reveals that the reduction rate of root activity was greater during the vegetative growth stage than during the flowering bud differentiation stage (**Table 2**).

**Table 2:** Summary of two-way analysis of variance (ANOVA) for heat stress (HS) and growth stage (S) on stomatal density, length and width, leaf stomatal conductance (g_s_), leaf transpiration (E), root activity, and root dry mass (Data are presented in Figs. 2, 4, and 5), as well as on total daily transpiration (T_r_) and evapotranspiration (ET) during varied duration of heat stress (T_r___HS_, ET__HS_) and recovery phase (T_r___recovery_, ET_recovery).

Duration (d)	Factor	Stomatal density	Stomatal length	Stomatal width	*g_s_*	E	Root activity	Root dry mass	Total T_r___HS_	Total T_r___recovery_	Total ET__HS_	Total ET__recovery_
5	HS	n.s.	n.s.	**	***	***	*	n.s.	***	n.s.	***	**
	S	***	**	***	***	***	***	***	***	***	***	***
	HS × S	n.s.	n.s.	n.s.	n.s.	n.s.	n.s.	n.s.	n.s.	n.s.	*	n.s.
10	HS	*	**	*	***	***	***	n.s.	***	***	***	**
	S	**	***	**	**	***	***	***	***	***	***	***
	HS × S	n.s.	n.s.	n.s.	*	*	***	n.s.	*	*	*	*
15	HS	*	*	*	***	***	***	*	***	***	***	**
	S	*	***	***	*	***	***	***	***	***	***	***
	HS × S	n.s.	n.s.	n.s.	*	*	***	*	**	*	*	*
20	HS	***	**	***	***	***	***	***	***	***	***	**
	S	***	***	***	**	***	***	***	***	***	***	***
	HS × S	n.s.	n.s.	n.s.	*	*	*	*	*	**	*	*

n.s., *, **,*** indicate non-significance and significance at P ≤ 0.05, 0.01 or 0.001, respectively.

The response of root dry mass to heat stress was delayed than that of the root activity. The heat stress-induced decline of root dry mass appeared after lasting 15 days during the vegetative growth stage and 20 days during the flowering bud differentiation stage (**Figure 2**). With respect to CK, the root dry mass was reduced by 26.9% and 27.9% for the HS-V-15 and HS-V-20 conditions but by 15.0% for the HS-F-20 condition. The reduction of root dry mass was significantly lower during the vegetative growth stage than during the flowering bud differentiation stage (**Table 2**).

### 3.2 Response of leaf area to heat stress

**Figure 3:**
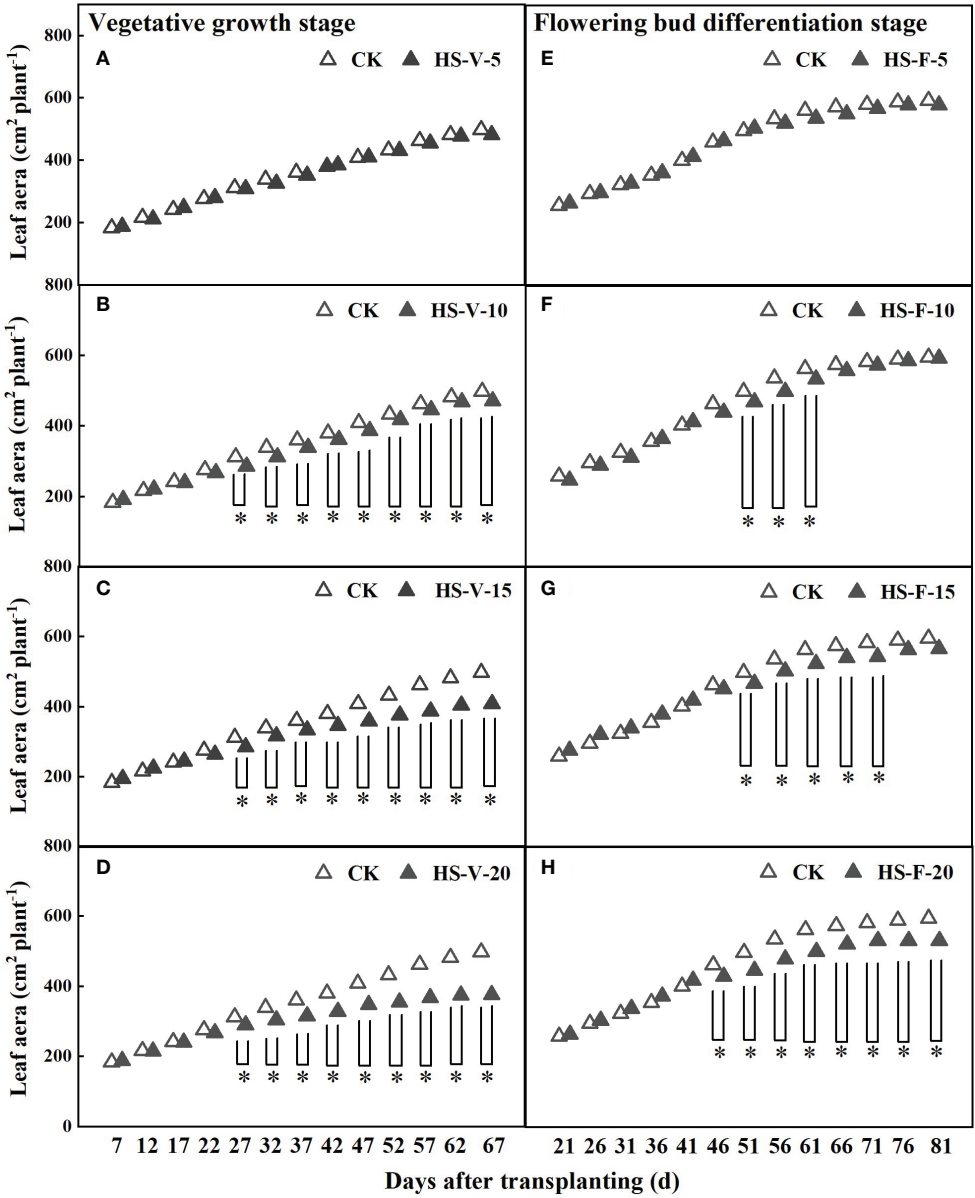
Dynamics of leaf area under different temperature treatments during the vegetative growth **(A–D)** and flowering bud differentiation stages **(E–H)**. CK is the control treatment, HS is the heat stress treatment. Values are the mean ± SD (n=3). *indicates the significant level at *P*<0.05.

Prolonged heat stress inhibited the leaf growth of gerbera (**Figure 3**). The leaf area of gerbera was significantly decreased after a 10-day-long heat stress. The leaf area of the HS-V-10, HS-V-15, and HS-V-20 conditions was decreased by 3.1%-8.3%, 4.2%-18.0%, and 7.1%-24.4%, respectively with respect to CK. However, the leaf area of the HS-F-10, HS-F-15, and HS-F-20 conditions was decreased by 0.5%-6.0%, 4.5%-6.3%, and 6.9%-10.6%, respectively compared to CK. Although the leaf area was still restricted during the recovery phase, the overall adverse effect of leaf area was low (**Figure 3**).

### 3.3 Effect of heat stress on leaf stomatal characteristics

**Figure 4:**
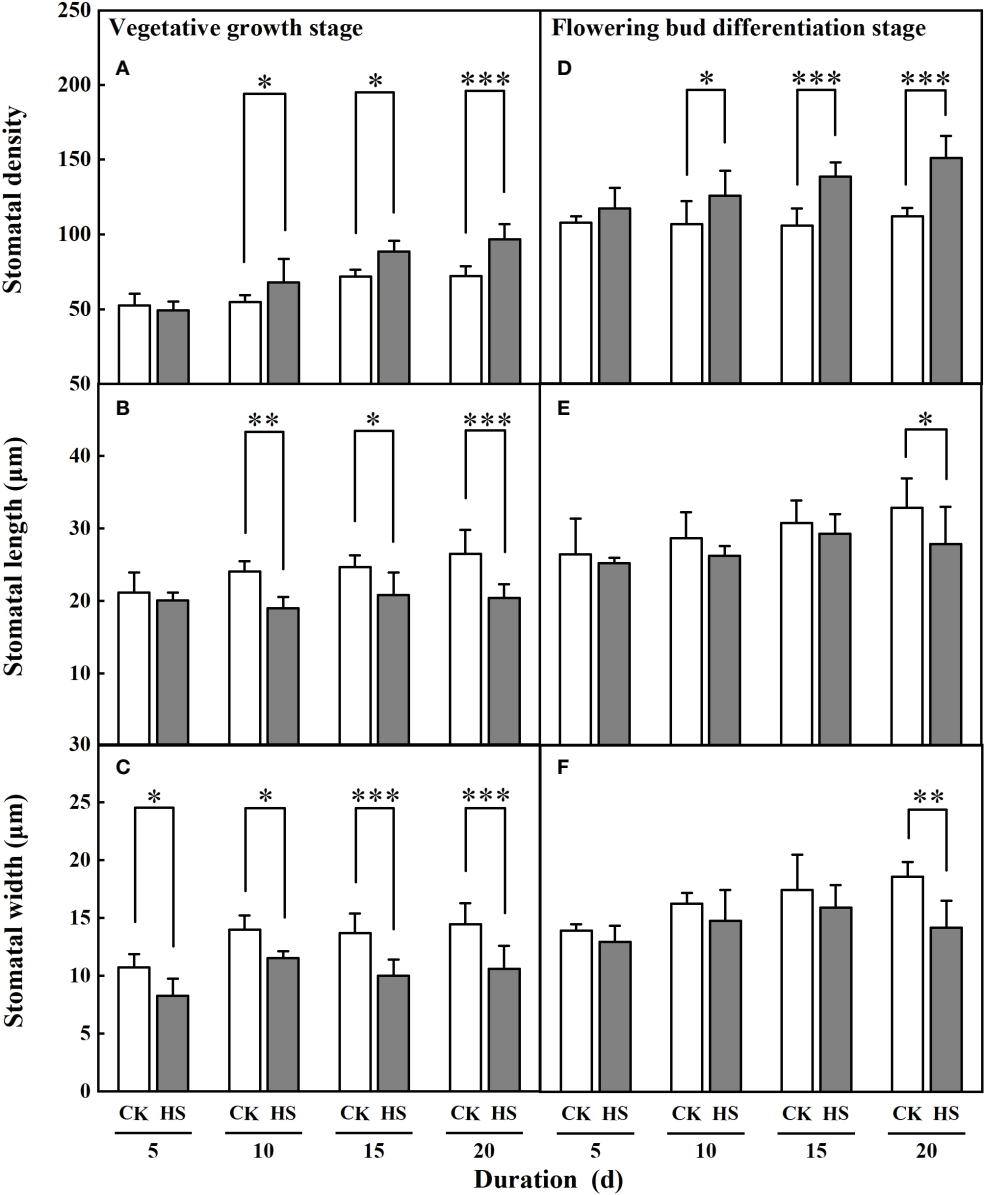
Response of stomatal density **(A, D)**, length **(B, E)** and width **(C, F)** of gerbera to heat stress (HS) during vegetative growth **(A–C)** and flowering bud differentiation stages **(D-F)**. CK is the control treatment. Values are the mean ± SD (n=5). *, **, and *** represent the significant level at *P*<0.05, *P*<0.01, and *P*<0.001, respectively.

**Figure 4** shows that the stomatal density and length significantly changed after a 10-day-long heat stress. During the vegetative growth stage, the stomata of leaves for the HS-V-10, HS-V-15, and HS-V-20 conditions was 24.3%, 23.1%, and 34.1% denser, respectively, compared to CK. Similarly, the stomatal length in these conditions was 21.3%, 15.6%, and 23.0% shorter, respectively. In addition, the stomatal width was significantly smaller (17.6%–26.9%) in all the heat stress conditions during the vegetative growth stage.

Similar to the vegetative growth stage, a significant increase in stomatal density during bud differentiation stage was observed after a 10-day-long heat stress. It was 17.7%, 31.1%, and 34.7% greater in HS-F-10, HS-F-15, and HS-F-20 conditions, respectively, compared to CK. However, the significant reduction of stomatal length and width was only observed for the HS-F-20 condition.

### 3.4 Response of leaf stomatal conductance and leaf transpiration to heat stress

**Figure 5:**
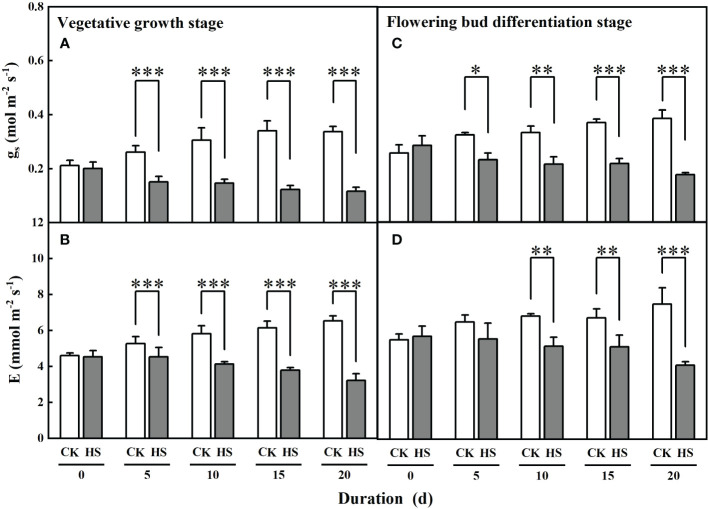
Effects of heat stress (HS) during the vegetative growth **(A, B)** and flowering bud differentiation stages **(C, D)** on leaf stomatal conductance (g_s_; **A, C**) and leaf transpiration (**E; B, D**) of gerbera. CK is the control treatment. Values are the mean ± SD (n = 3). *, **, and *** represent the significant level at *P*<0.05, *P*<0.01, and *P*<0.001, respectively.

**Figure 5** shows that the values of leaf stomatal conductance significantly decreased for all heat stress treatments during two growth stages. Compared to CK, leaf stomatal conductance was significantly smaller for all heat stress treatments during the vegetative growth stage (42.2%-65.4%) and flowering bud differentiation stage (28.5%-54.4%). Similarly, leaf transpiration was significantly reduced by 13.7%, 29.1%, 38.4%, and 50.7%, respectively, for the HS-V-5, HS-V-10, HS-V-15, and HS-V-20 conditions, and by 24.5%, 23.7%, and 45.3%, respectively, for the HS-F-10, HS-F-15, and HS-F-20 conditions relative to CK (**Figure 5**). These values suggest greater reduction rate of leaf stomatal conductance and leaf transpiration in the vegetative growth stage than the flowering bud differentiation stage (**Table 2**).

### 3.5 Effects of heat stress on transpiration and evapotranspiration

#### 3.5.1 Hourly scale

**Figure 6:**
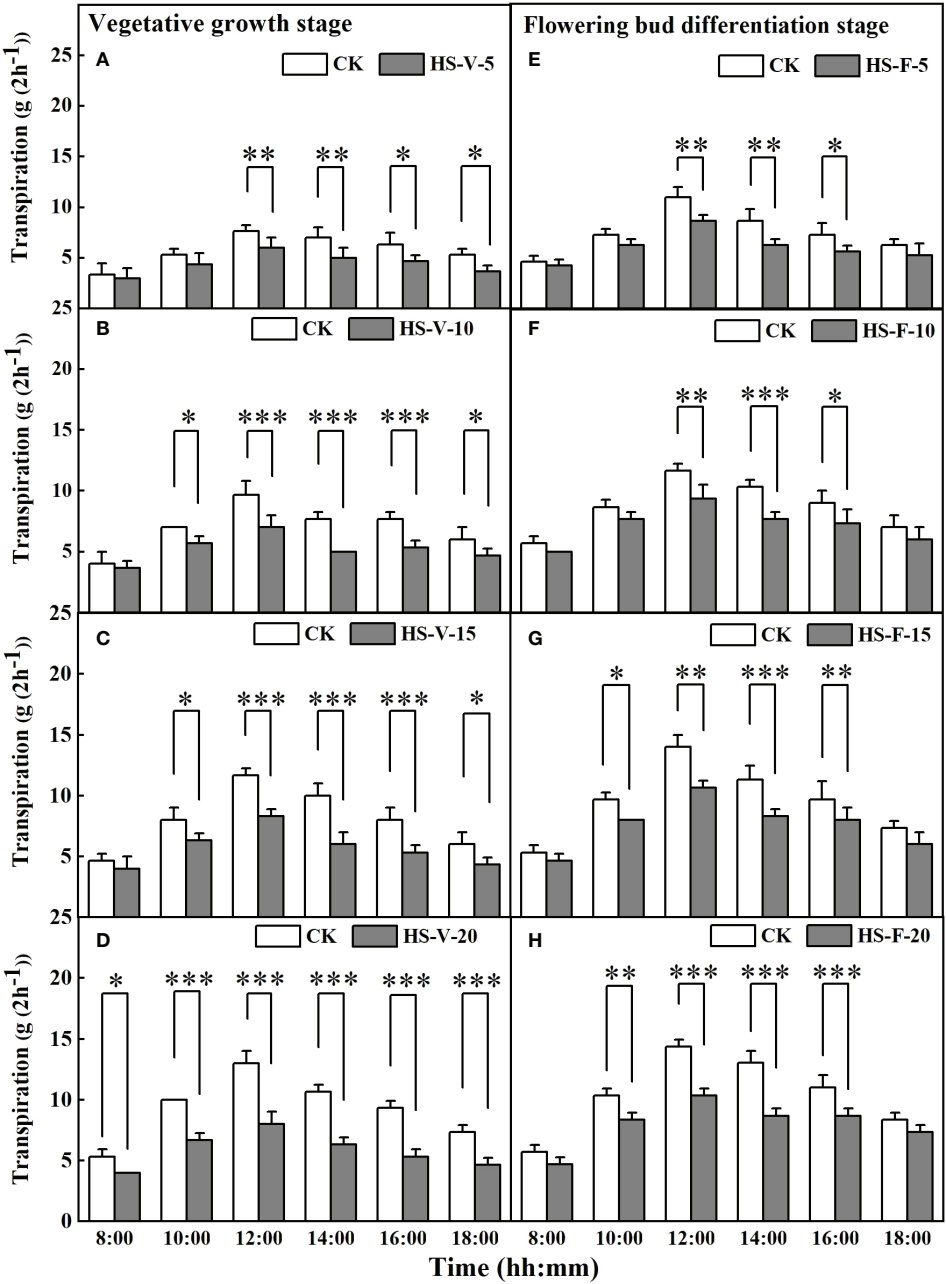
Diurnal variation of transpiration under different temperature treatments during the vegetative growth **(A–D)** and flowering bud differentiation stages **(E–H)**. *, **, and *** represent the significant level at *P*<0.05, *P*<0.01, and *P*<0.001, respectively. The error bars represent standard deviation (n=3).

**Figure 6** shows that heat stress significantly affected hourly transpiration rate in part of the daytime, except for the HS-V-20 condition (entire daytime). For instance, compared to CK, the hourly transpiration rate was decreased significantly by 21.7%-28.6% during the period 12:00-18:00 for the HS-V-5 condition, and by 19.0%-34.8% and 20.8%-40.0%, respectively, during the period 10:00-18:00 for the HS-V-10 and HS-V-15 conditions. Likewise, for the flowering bud differentiation stage, the hourly transpiration rate was reduced significantly by 21.2%-26.9% and 18.5%-25.8%, respectively, during the period 12:00-16:00 for the HS-F-5 and HS-F-10 conditions relative to CK; and by 17.2%-28.6% and 19.4%-33.3% respectively, during 10:00 and 16:00 for the HS-F-15 and HS-F-20 conditions. Specifically, the HS-V-20 condition significantly affected hourly transpiration over the entire daytime period with a reduction rate of 25.0%-42.3%.

**Figure 7:**
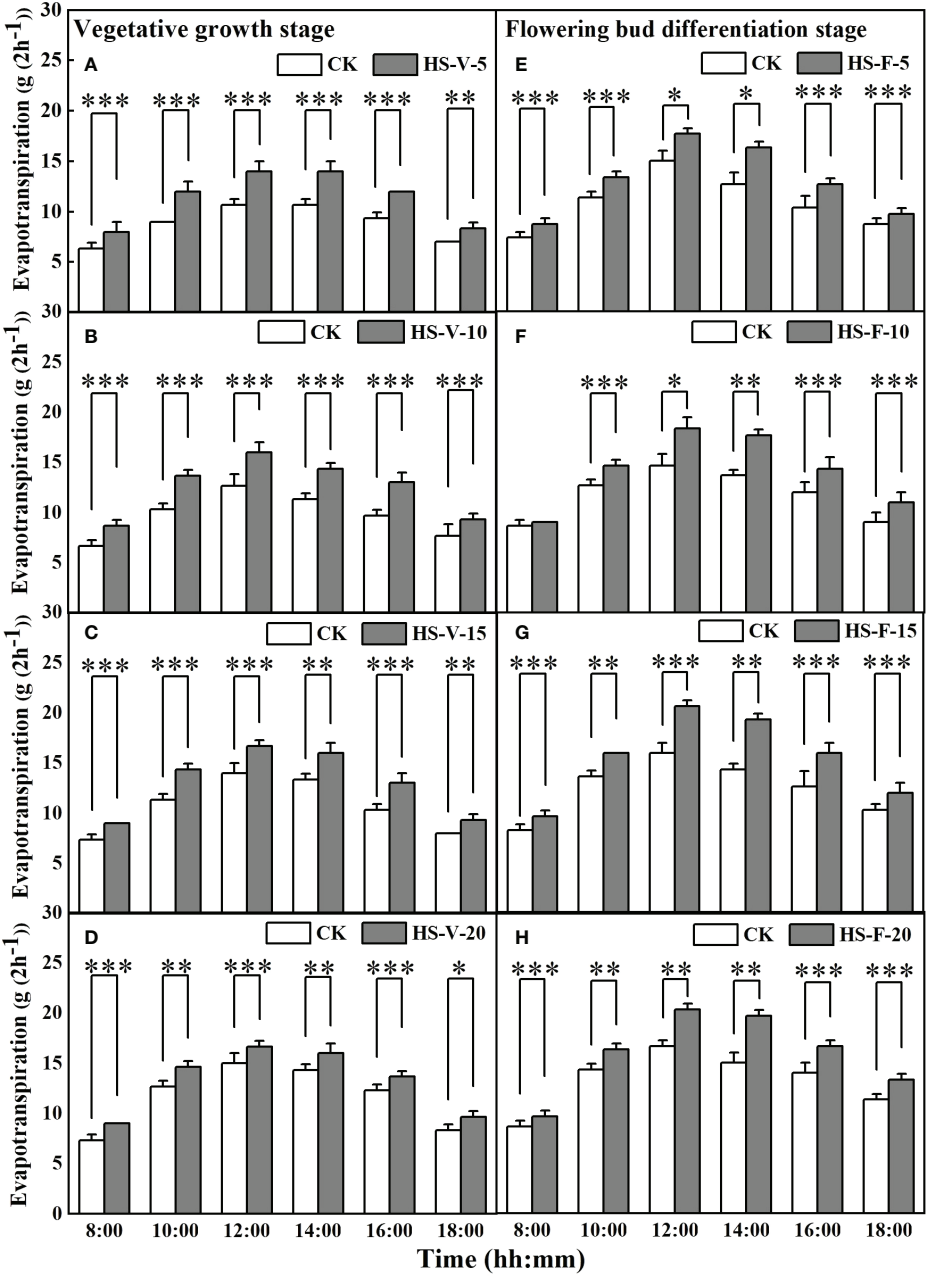
Diurnal variation of evapotranspiration under different temperature treatments during the vegetative growth **(A–D)** and flowering bud differentiation stages **(E–H)**. *, **, and *** represent the significant level at *P*<0.05, *P*<0.01, and *P*<0.001, respectively. The error bars represent standard deviation (n=3).

Interestingly, heat stress significantly increased hourly evapotranspiration rate for almost entire daytime hours (**Figure 7**). Compared to CK, the hourly evapotranspiration of the HS-V-5, HS-V-10, HS-V-15, and HS-V-20 conditions was increased by 22.2%-37.5%, 25.0%-35.7%, 19.1%-29.0%, and 18.2%-28.1%, respectively, during the period 8:00-18:00. Similarly, during daytime in the flowering bud differentiation stage, the hourly evapotranspiration was increased significantly by 23.5%-43.7% for the HS-F-5 condition except for 18:00; and by 25.0%-42.9%, 20%-40.5%, and 21.4%-35.9%, respectively, for the HS-F-10, HS-F-15, and HS-F-20 conditions except for 8:00.

#### 3.5.2 Daily scale

**Figure 8:**
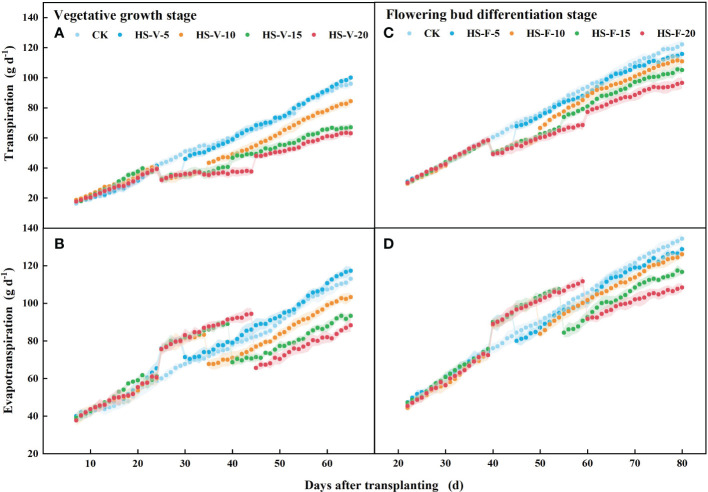
Variation of daily transpiration **(A, C)** and evapotranspiration **(B, D)** exposed to different temperature treatments during the vegetative growth **(A, B)** and flowering bud differentiation stages **(C, D)**. The shading areas of each line represent standard deviation.

There was no significant difference (*P* > 0.05) in the daily transpiration and evapotranspiration rate among all treatments before imposing heat stress (**Figure 8**). The HS-V-5, HS-V-10, HS-V-15, and HS-V-20 conditions decreased daily transpiration by 19.7%-22.2%, 18.9%-26.0%, 19.2%-27.3%, and 21.3%-31.8%, respectively, compared to CK. During the recovery phase, there was no significant difference in daily transpiration between the HS-V-5 condition and CK. However, daily transpiration could not recover after the end of heat stress in the other treatments, remaining 11.2%-14.4%, 14.9%-19.8%, and 21.2%-26.9% decrease for the HS-V-10, HS-V-15, and HS-V-20 conditions, respectively (**Figure 8**).

During the flower bud differentiation stage, daily transpiration was 12.8%-16.8%, 13.3%-16.9%, 12.1%-16.7%, and 13.6%-20.3% less for the HS-F-5, HS-F-10, HS-F-15, and HS-F-20 conditions, respectively, relative to CK. Plants of the HS-F-5 and HS-F-10 conditions exhibited a less than 10% reduction in daily transpiration during the recovery phase, while the reduction rate was 13.8%-16.3% and 14.8%-22.1% for the HS-F-15 and HS-F-20 conditions, respectively.

**Figure 9:**
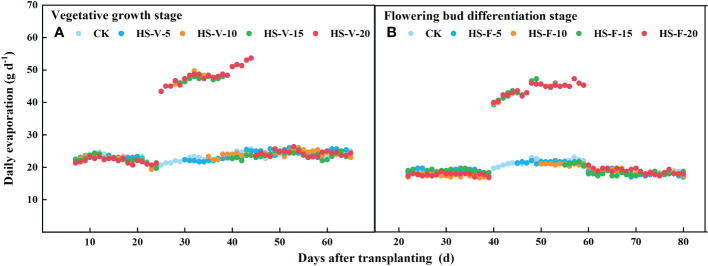
Variation of daily evaporation **(A, B)** exposed to different temperature treatments during the vegetative growth **(A)** and flowering bud differentiation stages **(B)**.

Different from daily transpiration, daily evapotranspiration was increased during the heat stress period (HS-V-5: 20.5%-24.3%; HS-V-10: 18.0%-24.5%; HS-V-15: 14.4%-23.7%; HS-V-20: 12.6%-22.8%). However, during the recovery phase, the daily evapotranspiration significantly reduced when the evaporation demand was almost the same (**Figure 9**). Compared to CK, the reduction rates of daily evapotranspiration during the recovery phase were greater under the HS-V-15 (11.2%~15.7%) and HS-V-20 (16.4%~22.7%) conditions than the HS-V-5 and HS-V-10 (<10%).

Similar to the vegetative growth stage, the daily evapotranspiration was increased by 13.5%-16.0%, 12.5%-15.9%, 11.0%-17.6%, and 8.4%-16.3%, respectively, for the HS-F-5, HS-F-10, HS-F-15, and HS-F-20 conditions during the heat stress period. During the recovery phase, the daily evapotranspiration was reduced by less than 10.0% for the HS-F-5 and HS-F-10 conditions and by 12.0%-14.6% and 11.1%-19.2% for the HS-F-15 and HS-F-20 conditions. **Table 2** also shows that heat stress occurred at different growth stages had significant effect on the total daily transpiration and evapotranspiration during the heat stress period and recovery phase.

## 4 Discussion

Heat stress is one of the main factors influencing crop evapotranspiration. As the increasing frequency and intensity of heat stress worldwide (IPCC, 2021), the effect of heat stress on evapotranspiration continues to receive more attentions. In this study, we showed that varied duration of heat stress at different growth stages had different effects on transpiration and evapotranspiration of gerbera.

Short-term heat stress affects the hourly transpiration in the period of high air temperature. For instance, the hourly transpiration was significantly decreased during the period 12:00-16:00 for the HS-V-5 condition (**Figure 6**), which is attributed to the limitation of stomatal width and stomatal conductance. High temperature triggers the water-saving mechanism of plants, which leads to partially closed stomata and smaller stomatal conductance, in turn preventing excessive water loss (Leuning, 1990; Damour et al., 2010; Medlyn et al., 2011). When the heat stress continued, the period of inhibited hourly transpiration was further prolonged. For instance, the hourly transpiration of the HS-V-10 condition was significantly decreased from 10:00 to 18:00. At the same time, a marked decline was also observed for root activity, stomatal size, and leaf area under the HS-V-10 condition compared to CK. High temperature lasting for a certain time significantly suppressed the root activity, which limited the root water uptake from soil and thus the water supply to aboveground organs (Nagel et al., 2009; Luo et al., 2020). The smaller stomatal size also suggests a smaller effective area for stomatal gas exchange (Bertolino et al., 2019). In addition, the heat stress gradually affects the development of leaf biomass, which in turn affects the transpiration. The declined root activity induced by heat stress limits the water and nutrients transport in plants, which inhibits the leaf growth, hence indirectly affecting the transpiration rate. Heat stress can also decrease the leaf area by inhibiting the cell division and elongation, directly reducing the plant evaporative surface (Schoppach and Sadok, 2013). In addition, it also affects protective enzymes and proteins, damaging leaf function, accelerating leaf senescence, and indirectly limiting transpiration (Balfagón et al., 2020). When heat stress lasted 15 days during the vegetative growth stage, a significant reduction in root biomass was observed. After 20 days of heat stress during the vegetative growth stage, the development of roots, leaves, and stomata was restricted, and inhibition of the transpiration by the HS-V-20 condition occurred in the entire daytime, and reached the maximum extent with respect to other conditions.

Heat stress at different growth stages also has varied effects on transpiration. Plants are more sensitive to heat stress at the vegetative growth stage than at the flowering bud differentiation stage (**Table 2**). For instance, compared to CK, heat stress markedly reduced daily transpiration by 18.9%-31.8% during the vegetative growth stage, whereas by 12.1%-20.3% during the flowering bud differentiation stage (**Figures 8A, C**). This different reduction rate of daily transpiration for heat stress at different growth stages was also supported by differences in the studied root, leaf, and stomata traits under heat stress (**Table 2**). Plants in the vegetative growth stage experience more extreme thermal environments than larger and older plants at high radiant energy input as a result of weak convection cooling potential (Villagarcía et al., 2007; Jagadish et al., 2021). In addition, the plants should have more heat tolerance in the flowering bud differentiation stage than the vegetative growth stage. Therefore, plants in the vegetative growth stage are more sensitive to and more stressed by heat stress.

There are also different responses of transpiration and evapotranspiration during the recovery phase. The daily transpiration of the HS-V-5 condition decreased by less than 10% in the early recovery stage, but by less than 5% in the late stage. However, the reduction of daily transpiration during the recovery phase increased with duration of heat stress. This may be because that short period (e.g., five days) of heat stress only limits the degree of stomatal conductance, which is easily resumed when the air temperature returned to normal. When heat stress lasted 10 days, the functions of the root activity and leaf area suffered from damage, and they could be recovered to a certain extent after the end of the heat stress. This may explain the small reduction of transpiration during the recovery phase. However, when the heat stress lasted 15 days, the plant biomass, especially the root and leaf, was inhibited, which directly affected the ability of water supply and transpiration during the recovery phase. Similar to the effect of heat stress on transpiration during different growth stages, plants could quickly recover after adversity during the flowering bud differentiation stage, resulting in a smaller accumulation of damage compared to that following adversity during the vegetative growth stage (**Table 2**).

Interestingly, heat stress increased daily evapotranspiration in this study (**Figures 8B, D**), and the increment rate was decreased as the duration of heat stress increased. Heat stress led to an increase in the potential evaporation rate (**Figure 1B**) as a result of increased VPD, which increased the evaporation by 100%-115% (**Figure 9**) during the heat stress period. This increased evaporation rate was higher than the rate of inhibition of the transpiration rate of gerbera, thus increasing evapotranspiration during the heat stress period. However, when the air temperature returned to normal, the daily evapotranspiration significantly decreased as a result of limited transpiration when the potential evaporation rate was almost the same. The results reported here is different from Qiu et al. (2022), who found that mean hourly evapotranspiration of flooded rice was reduced by 12% during the heat stress period, which may be attributed to that the rate of inhibition of the transpiration was greater than the increased evaporation rate.

## 5 Conclusions

We found that the response of the hourly transpiration to heat stress exhibited a temporal variation, with the duration of inhibition during the course of the day and the inhibition amplitude becoming greater as the duration of heat stress prolonged. The daily transpiration was considerably decreased by heat stress, and the amplitude of the decrease was greater for longer-lasting heat stress. Transpiration was more sensitive to heat stress during the vegetative growth stage than during the flowering bud differentiation stage. The daily transpiration could recover only when the heat stress lasted for a short period (e.g., five days) during the vegetative growth stage. Interestingly, the daily evapotranspiration markedly increased during heat stress period, as a result of disproportional effect of heat stress between evaporation and transpiration. However, during the recovery stage, daily evapotranspiration was reduced after heat stress. This study suggests that heat stress induced variation in evapotranspiration and its subsequent effect should be incorporated in evapotranspiration models and climate scenarios projections.

## Data availability statement

The original contributions presented in the study are included in the article/Supplementary Material. Further inquiries can be directed to the corresponding author.

## Author contributions

YJ: Data curation, Formal analysis, Methodology, Writing – original draft. ZY: Conceptualization, Methodology, Supervision, Writing – original draft. RQ: Methodology, Supervision, Validation, Formal analysis, Writing – review and editing. XG: Methodology, Writing – review and editing. EA: Methodology, Writing – review and editing. WH: Writing – review and editing. BC: Writing – review and editing. All authors contributed to the article and approved the submitted version.
